# Phosphodiesterase Inhibitors Suppress *Lactobacillus casei* Cell-Wall-Induced NF-**κ**B and MAPK Activations and Cell Proliferation through Protein Kinase A—or Exchange Protein Activated by cAMP-Dependent Signal Pathway

**DOI:** 10.1100/2012/748572

**Published:** 2012-05-03

**Authors:** Takekatsu Saito, Naotoshi Sugimoto, Kunio Ohta, Tohru Shimizu, Kaori Ohtani, Yuko Nakayama, Taichi Nakamura, Yashiaki Hitomi, Hiroyuki Nakamura, Shoichi Koizumi, Akihiro Yachie

**Affiliations:** ^1^Department of Pediatrics, Graduate School of Medical Science, Kanazawa University, Kanazawa 920-8640, Japan; ^2^Department of Physiology, Graduate School of Medical Science, Kanazawa University, Kanazawa 920-8640, Japan; ^3^Department of Microbiology, Graduate School of Medical Science, Kanazawa University, Kanazawa 920-8640, Japan; ^4^Department of Public Health, Graduate School of Medical Science, Kanazawa University, Kanazawa 920-8640, Japan; ^5^United Graduate School of Child Development, Kanazawa University, Kanazawa 920-8640, Japan

## Abstract

Specific strains of *Lactobacillus* have been found to be beneficial in treating some types of diarrhea and vaginosis. However, a high mortality rate results from underlying immunosuppressive conditions in patients with *Lactobacillus casei* bacteremia. Cyclic AMP (cAMP) is a small second messenger molecule that mediates signal transduction. The onset and progression of inflammatory responses are sensitive to changes in steady-state cAMP levels. *L. casei* cell wall extract (LCWE) develops arteritis in mice through Toll-like receptor-2 signaling. The purpose of this study was to investigate whether intracellular cAMP affects LCWE-induced pathological signaling. LCWE was shown to induce phosphorylation of the nuclear factor **κ**B (NF-**κ**B) and mitogen-activated protein kinase (MAPK) signaling pathways and cell proliferation in mice fibroblast cells. Theophylline and phosphodiesterase inhibitor increased intracellular cAMP and inhibited LCWE-induced cell proliferation as well as phosphorylation of NF-**κ**B and MAPK. Protein kinase A inhibitor H89 prevented cAMP-induced MAPK inhibition, but not cAMP-induced NF-**κ**B inhibition. An exchange protein activated by cAMP (Epac) agonist inhibited NF-**κ**B activation but not MAPK activation. These results indicate that an increase in intracellular cAMP prevents LCWE induction of pathological signaling pathways dependent on PKA and Epac signaling.

## 1. Introduction

Bacteria stimulate the innate immune system of a host and the release of inflammatory molecules such as cytokines and chemokines. Lipopolysaccharide (LPS) is an essential component of the cell wall of gram-negative bacteria, while peptidoglycan (PGN) is the major component of the cell wall in gram-positive bacteria. Exposure to LPS or PGN activates a number of inflammatory pathways, including the transcription factor NF-*κ*B and mitogen-activated protein kinase (MAPK) pathways [1, 2].

*Lactobacillus* is a gram-positive, rod-shaped bacterium ubiquitous in humans and inhabits the mouth and gastrointestinal tract. This organism is anaerobic, non-spore-forming, and nonmotile. Some *Lactobacillus* strains represent sources of beneficial organisms termed probiotics, which are defined as “live microorganisms which when administered in adequate amounts confer health benefits to the host” (FAO/WHO, 2001). *Lactobacillus* can modulate systemic inflammation, cell proliferation, and apoptosis, and such properties may be useful in immunomodulatory and cancer prevention strategies [3, 4]. However, approximately two-thirds of immunocompromised individuals with *Lactobacillus casei* bacteremia have underlying structural heart disease or have had a valvular or aortic prosthesis inserted; these patients are at risk for developing intravascular infection [5]. Additionally, Lehman et al. [6] reported that single intraperitoneal injection of a cell wall extract isolated from *L. casei* (LCWE) reproducibly leads to the development of arteritis. Recent studies suggesting signaling via Toll-like receptors (TLRs) in knockout mice indicate that TLR-2 signaling may play a role in LCWE-induced mouse arteritis [7]. However, the mechanism of pathogenesis in LCWE-induced mouse arteritis has not yet been elucidated.

The onset and progression of the inflammatory response are sensitive to changes in steady-state levels of the cyclic nucleotide cAMP [8]. cAMP is a small, second messenger molecule that mediates signal transduction initiated by ligand binding to G-protein coupled receptors such as prostaglandin and adrenergic receptors. Pharmacological manipulation of cyclic nucleotide phosphodiesterases (PDEs), which degrade the cyclic nucleotides cAMP and cGMP, provides a powerful strategy for regulating biological processes relayed by these intracellular second messengers [9].

In this study, we show that theophylline and an experimental, nonspecific PDE inhibitor attenuated LCWE-induced pathological signal transductions and cell proliferation in a protein kinase A (PKA)—and exchange protein activated by cAMP (Epac)-dependent manner.

## 2. Materials and Methods

### 2.1. Chemicals

Theophylline, isobutylmethylxanthine (IBMX), and Dulbecco's modified Eagle's medium (DMEM) were obtained from Wako Pure Chemical Industries, Ltd. (Osaka, Japan). 8-Bromoadenosine-3,5-cyclic monophosphate (8-Br-cAMP) and 8-(4-chlorophenylthio)-2-*O*-methyladenosine-3,5-cyclic monophosphate sodium salt (8-CPT-cAMP, Epac activator) were obtained from Biaffin GmbH & Co KG (Kassel, Germany). H89 was obtained from Calbiochem (La Jolla, CA). Fetal bovine serum (FBS) was obtained from Invitrogen Corporation (Carlsbad, CA). Anti-phospho-specific NF-*κ*B p65 (Ser536), anti-phospho-specific p44/42 MAPK (Thr202/Tyr204), anti-*β*-actin, horseradish-peroxidase-(HRP-) linked anti-rabbit IgG, and anti-mouse IgG were purchased from Cell Signaling Technology, Inc. (Danvers, MA). Anti-phospho-specific VASP (Ser157) was obtained from Calbiochem (La Jolla, CA).

### 2.2. *L. casei* Cell Wall Fragments

Group B *L. casei* cell wall fragments (LCWE) were obtained from *L. casei* (ATCC 11578) as described [6]. Briefly, the bacteria were disrupted by overnight incubation in 4% sodium dodecylsulphate (SDS) in twice their packed volume. Cell wall fragment preparations were sonicated for 2 h. During sonication, cell wall fragments were maintained at 4°C. After sonication, cell wall fragments were centrifuged for 1 h at 20,000 ×g at 4°C, and the supernatant was retained.

### 2.3. Cell Culture

NIH 3T3 mouse fibroblast cells were provided by Dr. Komine (Kanazawa University). The cells were maintained in DMEM containing 10% FBS at 37°C in a 5% CO_2_ incubator.

### 2.4. Cell Proliferation Assay

Cell proliferation was analyzed using the Cell Counting Kit 8 (Wako, Japan). NIH3T3 cells were seeded in 96-well plates at a density of 1 × 10^3^ cells/well. After a 24 h incubation, the cells were treated with LCWE, theophylline, IBMX, 8-Br-cAMP, or 8-CPT-cAMP for 72 h. Next, the cells were incubated with 10 *μ*L WST-8 for 2 h. Absorbance of the colored formazan product produced by mitochondrial dehydrogenases in metabolically active cells was recorded at 450 nm as the background value. Cell proliferation was expressed as a percentage of absorbance obtained in treated wells relative to that in untreated (control) wells.

### 2.5. NF-*κ*B and p44/42 MAPK (ERK) Activity Assays

To determine the effect of phosphodiesterase inhibitors or cAMP analogs on NF-*κ*B and MAPK (ERK) activities, we investigated phospho-NF-*κ*B and phospho-MAPK (ERK) levels using various methods. Increases in phospho-NF-*κ*B and phospho-MAPK (ERK) levels indicate the amount of NF-*κ*B and MAPK (ERK) signaling activation. NIH3T3 cells were incubated in DMEM containing serum for 24 h and treated with theophylline (10 mM), IBMX (1 *μ*M), 8-Br-cAMP (1 mM), or 8-CPT-cAMP (3 *μ*M) for 10 min with or without H89 (5 *μ*M). Western blotting analyses were performed using phospho-NF-*κ*B p65 (Ser536) antibody and phospho-p44/42 MAPK (Thr202/Tyr204) antibody.

### 2.6. Western Blotting Analysis

Western blotting was performed as described previously [10]. Briefly, proteins were extracted from cells, and protein concentrations were determined using a protein assay. Equal amounts of protein were separated using 10% sodium dodecyl sulfate-polyacrylamide gel electrophoresis (SDS-PAGE). Resolved proteins were transferred onto polyvinylidene fluoride (PVDF) membranes, which were incubated with primary antibodies (1 : 1000), followed by incubation with HRP-linked secondary antibodies (1 : 2000). The blots were developed using Immobilon Western Chemiluminescence HRP Substrate (Millipore, Billerica, MA).

### 2.7. RT-PCR Analysis

To evaluate the expression pattern of Toll-like receptor-2 (TLR-2) mRNA in the cells, reverse transcription-mediated polymerase chain reaction (RT-PCR) was performed as follows. Briefly, RNA was extracted from the cells and reverse-transcribed by using the reverse transcriptase ReverTra Ace (TOYOBO, Tokyo, Japan). PCR-based subtype-specific gene amplification for TLR-2 and *β*-actin was performed with LA Taq (Takara, Tokyo, Japan) using the sets of primers as follows: 5′-gagtggtgcaagtatgaact-3′ and 5′-ttgcagaagcgctggggaat-3′ for TLR-2 and 5′-atggtgggtatgggtcagaag-3′ and 5′-ctggggtgttgaaggtctcaa-3′ for *β*-actin.

### 2.8. Statistical Analysis

Data are presented as means ± SEM from at least 3 independent experiments. Statistical analyses were performed using ANOVA followed by Dunnett's test, and results were considered statistically significant when *P* < 0.05.

## 3. Results

### 3.1. LCWE and PGN Activate NF-*κ*B, ERK, and Cell Proliferation

**Figure 1:**
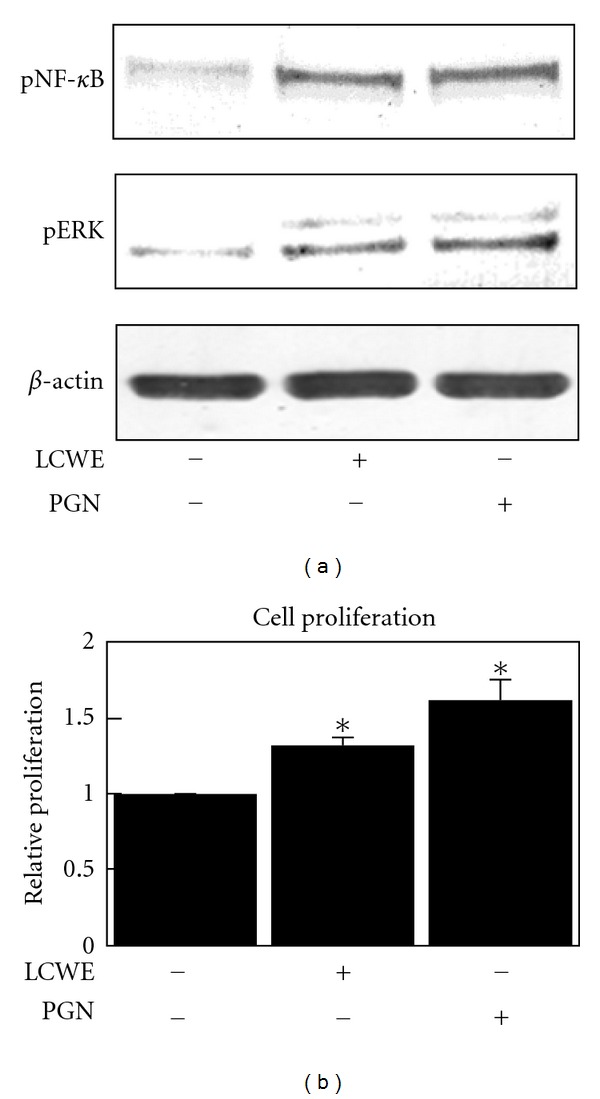
Changes in the levels of phospho-NF-*κ*B and phospho-ERK (a) and change in cell proliferation (b) in NIH3T3 mouse fibroblast cells after treatment with LCWE (10 *μ*g/mL) or PGN (10 *μ*g/mL). Each column represents the mean ± SEM. *P < 0.01  versus untreated controls.

Peptidoglycan (PGN) is a major component in the cell wall of gram-positive bacteria. LCWE was isolated from the gram-positive bacteria *L. casei*. First, we investigated whether LCWE, as well as PGN, induced the activation of NF-*κ*B and MAP kinase signaling pathways, *that is*, phosphorylation of NF-*κ*B (p65) and ERK1/2, respectively, in NIH 3T3 fibroblast cells. As shown in Figure 1(a), LCWE (10 *μ*g/mL) as well as PGN (10 *μ*g/mL) significantly increased the phosphorylation of NF-*κ*B and ERK, indicating that LCWE and PGN activate the NF-*κ*B and ERK signaling pathways in NIH 3T3 fibroblast cells. LCWE and PGN also accelerated cell proliferation (Figure 1(b)).

### 3.2. Theophylline Inhibits LCWE-Induced Phosphorylation of NF-*κ*B and ERK As Well As Cell Proliferation

**Figure 2:**
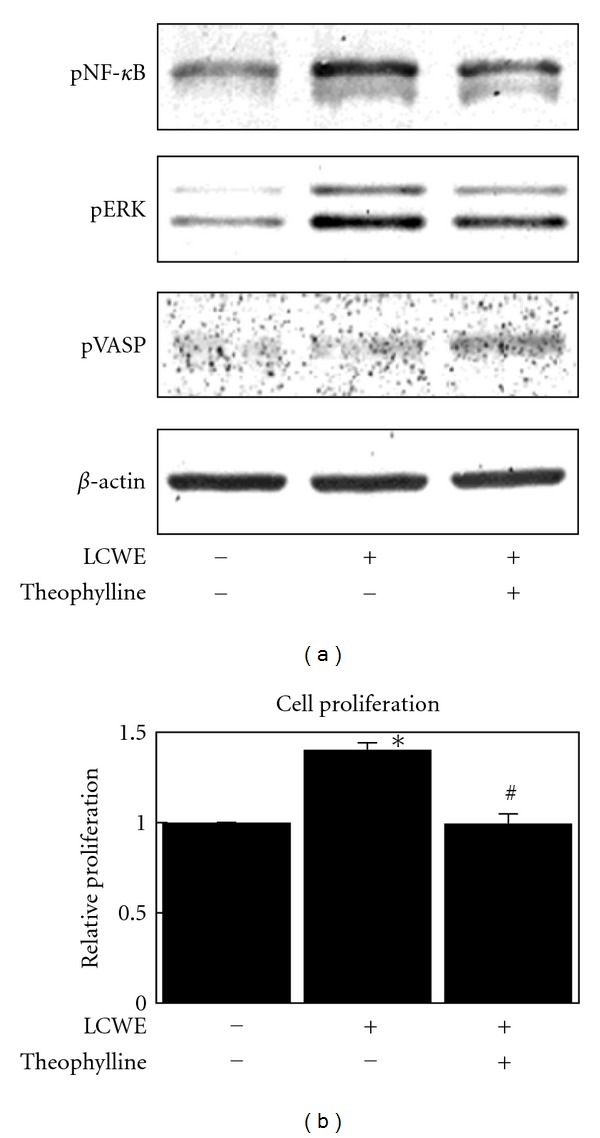
Changes in the levels of phospho-NF-*κ*B, phospho-ERK, and phospho-VASP (a) and change in cell proliferation (b) in NIH3T3 mouse fibroblast cells after treatment with LCWE (10 *μ*g/mL) or a combination of theophylline (10 mM) and LCWE (10 *μ*g/mL). Each column represents the mean ± SEM. *P < 0.01  versus untreated controls. ^#^ *P* < 0.01  versus LCWE treatment group.

Theophylline is a methylxanthine drug used in therapy for asthma, and a weak and nonselective inhibitor of phosphodiesterases (PDEs), which break down cyclic nucleotides in the cell, leading to an increase in intracellular cAMP concentrations [11, 12]. Vasodilator-stimulated phosphoprotein (VASP) is a critical factor in regulating actin dynamics; increased levels of phosphorylated VASP are related to increased levels of intracellular cAMP [13, 14]. Expression of phosphorylated VASP at serine-157 was upregulated after theophylline treatment (10 mM), indicating that theophylline increased intracellular cAMP concentration under this condition (Figure 2(a)). LCWE-induced NF-*κ*B and ERK phosphorylation were significantly inhibited by theophylline, suggesting that theophylline prevents LCWE-induced NF-*κ*B and ERK activation (Figure 2(a)). As shown in Figure 2(b), theophylline completely prevented LCWE-induced cell proliferation.

### 3.3. PDE Inhibitor IBMX Inhibits LCWE-Induced Phosphorylation of NF-*κ*B and ERK As Well As Cell Proliferation

**Figure 3:**
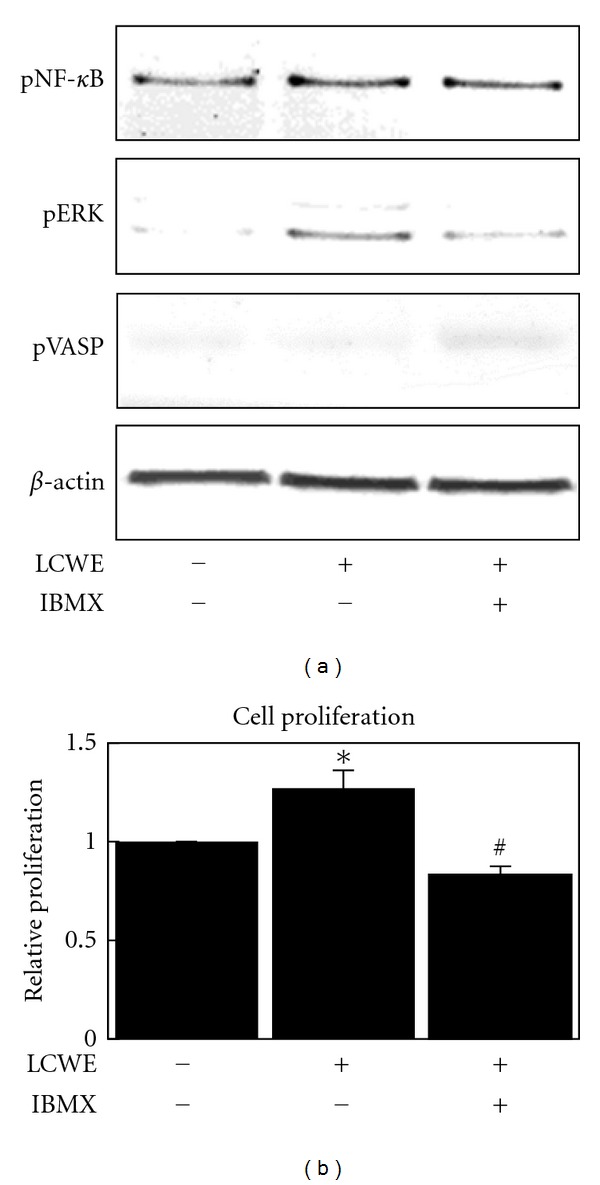
Changes in the levels of phospho-NF-*κ*B, phospho-ERK, and phospho-VASP (a) and change in cell proliferation (b) in NIH3T3 mouse fibroblast cells after treatment with LCWE (10 *μ*g/mL) or a combination of IBMX (1 *μ*M) and LCWE (10 *μ*g/mL). Each column represents the mean ± SEM. *P < 0.01  versus untreated controls. ^#^ *P* < 0.01  versus LCWE treatment group.

Isobutylmethylxanthine (IBMX) is a nonselective PDE inhibitor that is stronger than theophylline. Pretreating NIH3T3 mouse fibroblast cells for 30 min with IBMX at a concentration of 1 *μ*M led to a marked increase in VASP phosphorylation (Figure 3(a)), implying that IBMX significantly increases intracellular cAMP concentration.  Figure 3(b) shows that LCWE-induced NF-*κ*B and ERK phosphorylation were significantly inhibited by IBMX. Moreover, IBMX completely abolished LCWE-induced cell proliferation (Figure 3(b)).

### 3.4. IBMX Inhibits LCWE-Induced Expression of TLR-2

**Figure 4:**
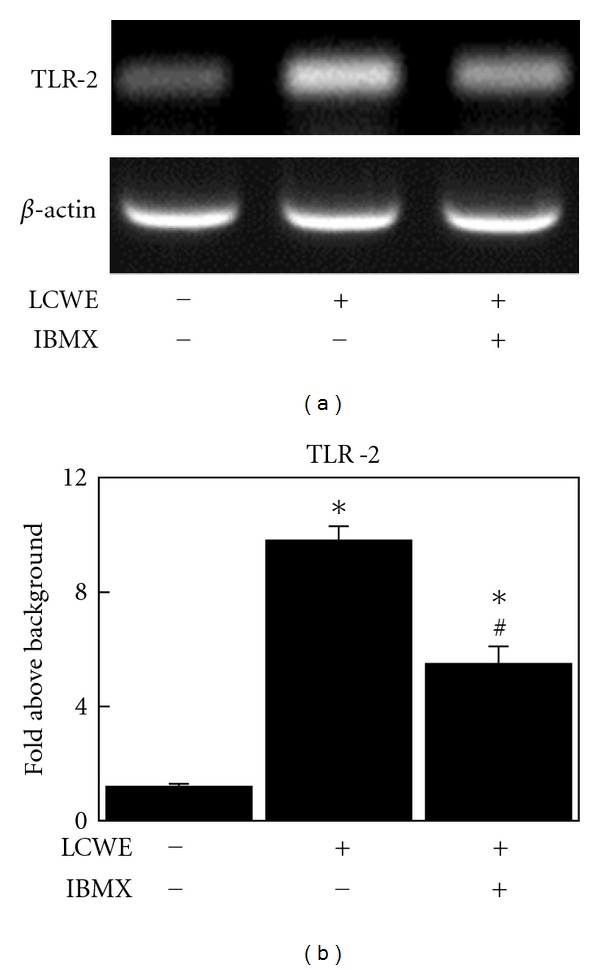
Change in the expression of TRL-2 (a, b) in NIH3T3 mouse fibroblast cells after treatment with LCWE (10 *μ*g/mL) or a combination of IBMX (1 *μ*M) and LCWE (10 *μ*g/mL). Each column represents the mean ± SEM. *P < 0.01  versus untreated controls. ^#^ *P* < 0.01  versus LCWE treatment group.

Expression of TLR-2, the primary LCWE receptor [7], was investigated in NIH3T3 mouse fibroblast cells using RT-PCR. The intensity of the TLR-2 band increased 16 h after LCWE treatment (Figure 4). However, pretreatment with IBMX for 30 min before LCWE stimulation significantly inhibited LCWE-induced expression of TLR-2 (Figure 4).

### 3.5. LCWE-Induced Inhibition of NF-*κ*B Phosphorylation by IBMX Is Independent of PKA, While LCWE-Induced Inhibition of ERK Activation by IBMX Is Dependent on PKA

**Figure 5:**
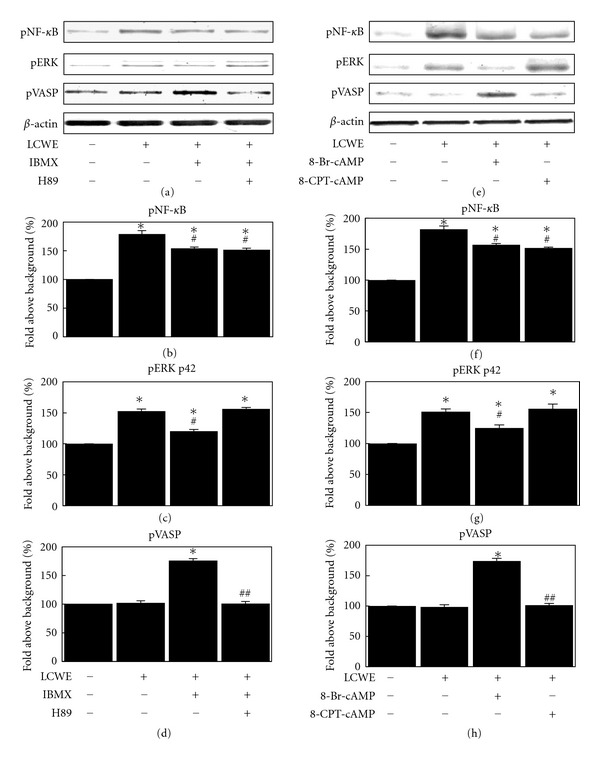
Change in the levels of phospho-NF-*κ*B (a, b), phospho-ERK (a, c), and phospho-VASP (a, d) in NIH3T3 mouse fibroblast cells after treatment with LCWE (10 *μ*g/mL), a combination of IBMX (1 *μ*M) and LCWE (10 *μ*g/mL), or a combination of IBMX (1 *μ*M), LCWE (10 *μ*g/mL), and H89 (5 *μ*M). Change in the levels of phospho-NF-*κ*B (e, f), phospho-ERK (e, g), and phospho-VASP (e, h) in NIH3T3 mouse fibroblast cells after treatment with LCWE (10 *μ*g/mL), a combination of 8-Br-cAMP (1 mM) and LCWE (10 *μ*g/mL), or a combination of 8-CPT-cAMP (5 *μ*M) and LCWE (10 *μ*g/mL). Each column represents the mean ± SEM. *P < 0.05  versus untreated controls. ^#^ *P* < 0.05  versus LCWE treatment group. ^##^ *P* < 0.01  versus LCWE and IBMX treatment group.

We subsequently examined whether the inhibitory effects of PDE inhibitor are due to PKA activation. For these studies, we examined the ability of the PKA inhibitor H89 to abrogate the ability of IBMX to inhibit LCWE-induced phosphorylation of NF-*κ*B and ERK. H89, which blocks the catalytic ATP binding site of PKA, was used at a concentration of 5 *μ*M, resulting in the inhibition of PKA-induced phosphorylation of VASP as shown in Figures 5(a) and 5(d). Pretreating cells with H89 blocked the ability of IBMX to inhibit LCWE-induced ERK phosphorylation, but not LCWE-induced NF-*κ*B phosphorylation (Figure 5). These results indicated that PDE inhibitor-mediated prevention of ERK is dependent on PKA activation, but PDE inhibitor-mediated inhibition of NF-*κ*B phosphorylation may be independent of PKA activation. 

### 3.6. cAMP Inhibits LCWE-Induced NF-*κ*B Activation via an Epac-Independent Signaling Pathway

PKA has long been thought to be the primary effector of cAMP in eukaryotic cells. However, Epac has recently been discovered to also function as a target for cAMP [15]. Epac is a guanine nucleotide exchange protein for the small GTPases Rap and has been shown to control many cellular processes previously attributed to PKA. Because we observed no role for PKA signaling in PDE inhibitor inhibition of NF-*κ*B phosphorylation in our studies, we shifted our focus to the potential involvement of Epac.

To further examine the potential signaling role of Epac, we examined the ability of the Epac-selective agonist 8-(4-chlorophenylthio)-2-*O*-methyladenosine-3,5-cyclic monophosphate sodium salt (8-CPT-cAMP) to inhibit LCWE-induced phosphorylation of NF-*κ*B. 8-CPT-cAMP is a nonhydrolyzable cAMP analog that activates Epac with greater potency than PKA [16]. Previous studies have shown that concentrations of 8-CPT-cAMP as low as 3 *μ*M effectively activate Epac in studies using cultured cells [17]. In our experiments, we observed that pretreating NIH3T3 cells with 3 *μ*M of 8-CPT-cAMP appreciably inhibited LCWE-induced phosphorylation of NF-*κ*B (Figures 5(e) and 5(f)). However, 3 *μ*M of 8-CPT-cAMP failed to significantly inhibit LCWE-induced phosphorylation of ERK (Figures 5(e) and 5(g)). 

The cAMP analog 8-bromoadenosine-3,5-cyclic monophosphate (8-Br-cAMP) induced inhibition of NF-*κ*B and ERK phosphorylation by LCWE (Figures 5(e) and 5(g)) similar to the level of IBMX inhibition of LCWE-induced phosphorylation of NF-*κ*B and ERK. These results strongly indicate that Epac signaling, but not PKA, is involved in mediating the inhibition of LCWE-induced phosphorylation of NF-*κ*B provided by the PDE inhibitor or by direct cAMP elevation.

### 3.7. cAMP Inhibits LCWE-Induced Cell Proliferation Depending on Epac Pathways

**Figure 6:**
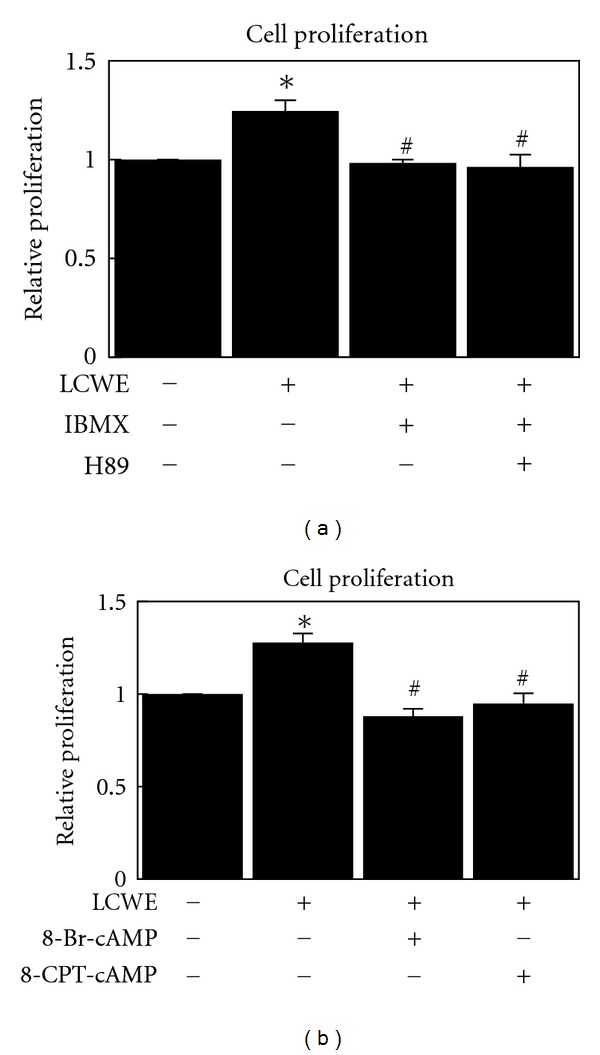
Change in cell proliferation after treatment with LCWE (10 *μ*g/mL), a combination of IBMX (1 *μ*M) and LCWE (10 *μ*g/mL), or a combination of IBMX (1 *μ*M), LCWE (10 *μ*g/mL), and H89 (5 *μ*M) (a) in NIH3T3 mouse fibroblast cells. Change in cell proliferation after treatment with LCWE (10 *μ*g/mL), a combination of 8-Br-cAMP (1 mM) and LCWE (10 *μ*g/mL), or a combination of 8-CPT-cAMP (5 *μ*M) and LCWE (10 *μ*g/mL) (b) in NIH3T3 mouse fibroblast cells. Each column represents the mean ± SEM. *P < 0.05  versus untreated controls. ^#^ *P* < 0.05  versus LCWE treatment group.

Next, we examined the ability of the PKA inhibitor H89 to abrogate the ability of IBMX to inhibit LCWE-induced cell proliferation. In Figure 6, H89 failed to inhibit IBMX-mediated prevention of LCWE-induced cell proliferation. The Epac activator 8-CPT-cAMP prevented LCWE-induced cell proliferation (Figure 6). These results indicated that cAMP inhibition of LCWE-induced cell proliferation is dependent on the Epac pathway.

## 4. Discussion

We showed that intracellular cAMP promotes antiproliferative and anti-inflammatory effects in LCWE-stimulated fibroblast cells via the modulation of NF-*κ*B and MAPK signaling. Our results show that intracellular cAMP induces the downregulation of NF-*κ*B and MAPK signaling dependent on Epac and PKA, respectively.

Specific strains of *Lactobacillus* have been found to be beneficial for treating certain types of diseases [3, 4]. However, *L. casei* bacteremia-induced high mortality rates have been explained based on underlying immunosuppressive conditions of the patients [5]. Thus, preventive strategies should be designed to reduce the pathological effects caused by lactobacilli.

Peptidoglycan is a characteristic cell wall component of gram-positive bacteria that can stimulate inflammatory signaling in several types of cells. In our study, we found that peptidoglycan potently induces NF-*κ*B and ERK phosphorylation in mouse fibroblast cells (Figure 1(a)). Additionally, it induces cell proliferation (Figure 1(b)). LCWE is composed of peptidoglycan and associated uncharged polysaccharides [18]. It was also found to phosphorylate NF-*κ*B and ERK at similar levels to those induced by PGN (Figure 1(a)). LCWE as well as PGN stimulated cell proliferation (Figure 1(b)). These results indicate that LCWE may regulate signal transduction through PGN receptors, specifically TLR-2 [7]. Interestingly, we observed that LCWE enhances TLR-2 mRNA expression in NIH3T3 cells (Figure 4). The regulation of the receptor by the ligand should be examined in future studies.

The NF-*κ*B signal pathway plays a crucial role in a variety of physiological and pathological events, including inflammation, immune responses, and apoptosis. In the canonical pathway, NF-*κ*B proteins are bound to inhibitory molecules (I*κ*Bs) and are sequestered in the cytoplasm in an inactive state. When cells are stimulated by the appropriate factors, the I*κ*B kinase (IKK) complex, containing catalytically active IKK*α* and IKK*β* and a regulatory scaffold protein IKK*γ*/NEMO, phosphorylates I*κ*B, leading to its ubiquitination and proteasomal destruction. NF-*κ*B is subsequently phosphorylated and released from inhibition to enter the nucleus and can either repress or activate gene transcription, including TLR-2 [19]. Our study showed that theophylline and IBMX, PDE inhibitors, have an inhibitory effect on LCWE-induced NF-*κ*B phosphorylation (Figures 2 and 3). These results suggest that PDE inhibitors may inhibit the TLR-2 receptor expression induced by LCWE (Figure 4).

Mitogen-activated protein kinases (MAPKs) such as ERK1/2, JNK1/2, and p38 play key roles in the transduction of extracellular signals to result in a cellular response. ERK1/2 can be activated by PGN [20]. In our study, we found that LCWE potently induces ERK1/2 activation in mouse fibroblast cells at similar levels to those induced by PGN (Figures 1(a), 1(b), and 3(a)). Additionally, our study described that theophylline and IBMX inhibited LCWE-induced ERK1/2 activation (Figures 2 and 3).

Elevated levels of cAMP in the cell lead to activation of different cAMP targets, including protein kinase A (PKA) and the exchange protein directly activated by cAMP (Epac). The 2 families of cAMP effectors provide a mechanism for precise and integrated control of cAMP signaling pathways in a spatial and temporal manner. PKA and Epac may act independently, converge synergistically, or oppose each other in regulating a specific cellular function [15]. In this study, we showed that PKA is responsible for cAMP-dependent VASP phosphorylation and ERK1/2 dephosphorylation, but not cAMP-dependent NF-*κ*B dephosphorylation (Figure 5). We also suggest that Epac is responsible for cAMP-dependent NF-*κ*B dephosphorylation (Figure 5), confirming that cAMP prevents LPS-induced IL-1*β* production through Epac [21]. Moreover, in this study, we showed that Epac plays a key role in cAMP-induced inhibition of cell proliferation (Figure 6). In our recent study, cAMP was shown to inhibit the activity of Akt through Epac-PTEN pathway activation and in turn inhibit cell proliferation in glial cells and osteosarcoma cells [17, 22]. In this study, theophylline and IBMX fully inhibited cell proliferation (Figures 2(b) and 3(b)) and Akt activation (data not shown). Theophylline and IBMX may inhibit cell proliferation through cAMP-induced Epac activation.

Our results provide a new insight into preventing cAMP action in LCWE-induced-pathological signaling. However, cAMP-induced inhibition of LCWE signaling in vivo is not understood. Further studies are necessary to determine the mechanism underlying cAMP effect on probiotics in vivo.

In conclusion, we showed that intracellular cAMP prevents LCWE-induced pathological signal transduction and cell proliferation. By understanding these signal transduction pathways, we can design preventive strategies for reducing the pathological effects caused by lactobacilli.

## References

[1] Xu Z, Dziarski R, Wang Q, Swartz K, Sakamoto KM, Gupta D (2001). Bacterial peptidoglycan-induced tnf-*α* transcription is mediated through the transcription factors Egr-1, Elk-1, and NF-*κ*B. *Journal of Immunology*.

[2] Dziarski R, Jin YP, Gupta D (1996). Differential activation of extracellular signal-regulated kinase (ERK) 1, ERK2, p38, and c-Jun NH2-terminal kinase mitogen-activated protein kinases by bacterial peptidoglycan. *Journal of Infectious Diseases*.

[3] Kato I, Endo-Tanaka K, Yokokura T (1998). Suppressive effects of the oral administration of Lactobacillus casei on type II collagen-induced arthritis in DBA/1 mice. *Life Sciences*.

[4] Sheil B, McCarthy J, O’Mahony L (2004). Is the mucosal route of administration essential for probiotic function? Subcutaneous administration is associated with attenuation of murine colitis and arthritis. *Gut*.

[5] Cannon JP, Lee TA, Bolanos JT, Danziger LH (2005). Pathogenic relevance of *Lactobacillus*: a retrospective review of over 200 cases. *European Journal of Clinical Microbiology & Infectious Diseases*.

[6] Lehman TJ, Walker SM, Mahnovski V, McCurdy D (1985). Coronary arteritis in mice following the systemic injection of group B *Lactobacillus casei* cell walls in aqueous suspension. *Arthritis and Rheumatism*.

[7] Rosenkranz ME, Schulte DJ, Agle LM (2005). TLR2 and MyD88 contribute to *Lactobacillus casei* extract-induced focal coronary arteritis in a mouse model of Kawasaki disease. *Circulation*.

[8] Moore AR, Willoughby DA (1995). The role of cAMP regulation in controlling inflammation. *Clinical and Experimental Immunology*.

[9] Mehats C, Andersen CB, Filopanti M, Jin SL, Conti M (2002). Cyclic nucleotide phosphodiesterases and their role in endocrine cell signaling. *Trends in Endocrinology and Metabolism*.

[10] Sugimoto N, Takuwa N, Yoshioka K, Takuwa Y (2006). Rho-dependent, Rho kinase-independent inhibitory regulation of Rac and cell migration by LPA1 receptor in Gi-inactivated CHO cells. *Experimental Cell Research*.

[11] Rabe KF, Magnussen H, Dent G (1995). Theophylline and selective PDE inhibitors as bronchodilators and smooth muscle relaxants. *European Respiratory Journal*.

[12] Hidi R, Timmermans S, Liu E (2000). Phosphodiesterase and cyclic adenosine monophosphate-dependent inhibition of T-lymphocyte chemotaxis. *European Respiratory Journal*.

[13] Comerford KM, Lawrence DW, Synnestvedt K, Levi BP, Colgan SP (2002). Role of vasodilator-stimulated phosphoprotein in PKA-induced changes in endothelial junctional permeability. *The FASEB Journal*.

[14] Loza MJ, Foster S, Peters SP, Penn RB (2006). Beta-agonists modulate T-cell functions via direct actions on type 1 and type 2 cells. *Blood*.

[15] Gloerich M, Bos JL (2010). Epac: defining a new mechanism for cAMP action. *Annual Review of Pharmacology and Toxicology*.

[16] Enserink JM, Christensen AE, de Rooij J (2002). A novel Epac-specific cAMP analogue demonstrates independent regulation of Rap1 and ERK. *Nature Cell Biology*.

[17] Sugimoto N, Miwa S, Ohno-Shosaku T (2011). Activation of tumor suppressor protein PTEN and induction of apoptosis are involved in cAMP-mediated inhibition of cell number in B92 glial cells. *Neuroscience Letters*.

[18] Shida K, Kiyoshima-Shibata J, Kaji R, Nagaoka M, Nanno M (2009). Peptidoglycan from *lactobacilli* inhibits interleukin-12 production by macrophages induced by *Lactobacillus casei* through toll-like receptor 2-dependent and independent mechanisms. *Immunology*.

[19] Komori M, Nakamura Y, Ping J (2011). *Pneumococcal* peptidoglycan-polysaccharides regulate toll-like receptor 2 in the mouse middle ear epithelial cells. *Pediatric Research*.

[20] Chen BC, Chang YS, Kang JC (2004). Peptidoglycan induces nuclear factor-*κ*B activation and cyclooxygenase-2 expression via Ras, Raf-1, and ERK in RAW 264.7 macrophages. *Journal of Biological Chemistry*.

[21] Xu XJ, Reichner JS, Mastrofrancesco B, Henry WL, Albina JE (2008). Prostaglandin E2 suppresses lipopolysaccharide-stimulated IFN-*β* production. *Journal of Immunology*.

[22] Miwa S, Sugimoto N, Shirai T (2011). Caffeine activates tumor suppressor PTEN in sarcoma cells. *International Journal of Oncology*.

